# A systematic review of the role of eculizumab in systemic lupus erythematosus-associated thrombotic microangiopathy

**DOI:** 10.1186/s12882-020-01888-5

**Published:** 2020-06-30

**Authors:** Rachael D. Wright, Fariba Bannerman, Michael W. Beresford, Louise Oni

**Affiliations:** 1 Department of Women’s and Children’s Health, Institute of Life Course and Medical Sciences, University of Liverpool, member of Liverpool Health Partners, Eaton Road, Liverpool, L12 2AP UK; 2 Library and Knowledge Service, Alder Hey Children’s NHS Foundation Trust, member of Liverpool Health Partners, Liverpool, UK; 3 Department of Paediatric Rheumatology, Alder Hey Children’s NHS Foundation Trust, member of Liverpool Health Partners, Liverpool, UK; 4 Department of Paediatric Nephrology, Alder Hey Children’s NHS Foundation Trust, member of Liverpool Health Partners, Liverpool, UK

**Keywords:** Lupus nephritis, Eculizumab, Complement, Thrombotic microangiopathy, Systematic review

## Abstract

**Background:**

Lupus nephritis (LN) is a severe consequence of systemic lupus erythematosus (SLE) that affects approximately 40% of patients. Pathogenic immune complexes that are characteristic of LN deposit in the kidney and activate immune mediated pathways including the complement system. Complete remission rates in LN are approximately 44% highlighting the need for new treatment strategies in these patients. Eculizumab is a fully humanised IgG2/IgG4 monoclonal antibody directed at C5 and thus prevents the formation of the terminal complement complex. Eculizumab is successfully used in atypical haemolytic uraemic syndrome (aHUS) and paroxysomal nocturnal haemoglobinuria (PNH) but it is not standardly used in LN. The aim of this project was to determine whether there is any role for eculizumab as adjunctive therapy in LN.

**Methods:**

Using a predefined search strategy on Ovid MEDLINE and EMBASE the literature was reviewed systematically to identify studies in which eculizumab had been used to treat patients with SLE. All patients were included that were treated with complement inhibitors. Favourable outcome in this study was defined as resolution of symptoms that led to treatment, discharge from hospital or recovery of renal function. Patients were excluded if there was no outcome data or if complement inhibition was unrelated to their SLE.

**Results:**

From 192 abstracts screened, 14 articles were identified, involving 30 patients. All SLE patients administered eculizumab were treated for thrombotic microangiopathy (TMA) secondary to LN diagnosed either histologically (66%) or as part of a diagnosis of aHUS (73%). 93% of patients had a favourable outcome in response to eculizumab treatment, of which 46% had a favourable outcome and successfully stopped treatment without relapse in symptoms during a median follow up of 7 months. Three patients (10%) reported adverse outcomes related to eculizumab therapy.

**Conclusions:**

Scientific evidence supports the involvement of complement in the pathogenesis of LN however the role of complement inhibition in clinical practice is limited to those with TMA features. This systematic review showed that in cases of LN complicated with TMA, eculizumab seems to be a very efficacious therapy. Further evidence is required to determine whether patients with refractory LN may benefit from adjunctive complement inhibition.

## Background

Systemic lupus erythematosus (SLE) is a severe, systemic autoimmune condition that is characterised by inflammation and organ damage. SLE patients experience a loss of tolerance towards self-antigens due to auto-reactive B cells that produce autoantibodies, particularly against double-stranded DNA (dsDNA) and nuclear antigens. These autoantibodies correlate closely with disease activity and are used for diagnostic and prognostic evaluation [1]. Approximately 9.7/10,000 people in the UK suffer from SLE and people of Black Caribbean ethnicity have a higher incidence and prevalence of the disease; they are also more likely to have worsened disease manifestations compared to people of White ethnicity [2]. Lupus nephritis (LN) is one of the most common and damaging manifestations of SLE that affects approximately 40% of patients and leads to end-stage renal failure within 5 years in approximately 10% [3]. Although evidenced-based therapeutic strategies are in place for LN (for instance use of disease modifying anti-rheumatic drugs (DMARDs) such as cyclophosphamide and mycophenolate mofetil [4]) and there are international recommendations for therapy (including the Single Hub and Access point for paediatric Rheumatology in Europe (SHARE) initiative recommendations [5]), many patients do not respond to therapy and the outcomes for LN are still poor (approximately 44% of patients will enter remission [6]).

LN occurs in response to pathogenic autoantibodies in SLE binding IgG to form immune complexes which deposit in the kidney and result in damage, in part through activation of the complement cascade and innate immune system [7–10]. Thrombotic microangiopathy (TMA) is a form of endothelial injury that can occur in the kidneys of 1–4% of LN patients [11] where it appears to correlate with the most severe clinical manifestations and is associated with a high mortality [12].

The complement system is part of the innate immune response involved in recognition and opsonisation of invading pathogens, it also plays important roles in maintaining homeostasis through clearance of apoptotic debris and immune complexes [13]. Complement plays an important role in LN – deficiencies in components of the complement system (C1q, C2 and C4) are prevalent in LN patients [14], particularly those that have a disease onset in childhood [15]. There is evidence to suggest that the alternative complement system can be pathogenically activated in SLE by the increased presence of immune complexes leading to overactivation of the terminal complement complex (C5b-9) and tissue damage [13, 16–18]. The kidney is particularly sensitive to immune complex deposition and thus complement-mediated damage [18, 19]. This is demonstrated through the increased levels of C5a and C5b-9 in the plasma of patients with active lupus nephritis [18].

Eculizumab is a recombinant fully humanised IgG2/IgG4 monoclonal antibody that binds C5 and thus prevents the formation of the terminal complement complex [20]. It is currently used successfully in the treatment of atypical haemolytic uraemic syndrome (aHUS) and paroxysomal nocturnal hemoglobinuria (PNH) [21, 22]. Eculizumab use is currently being explored in the treatment of other types of glomerulonephritis in which complement dysregulation is implicated including IgA nephropathy [23] and Shiga toxin-producing *Escherichia coli* (STEC-HUS) [24]. In view of its mode of action, eculizumab has also been considered for use in LN.

The aim of this project was to determine the role of eculizumab as adjunctive therapy in patients with LN. The objectives were to perform a systematic literature review using the PICOS framework – (P)articipants – all ages, sexes and ethnicities included, (I) ntervention – those who received complement inhibition therapy for their SLE, (C) omparison – before and after complement inhibition therapy, (O)utcome – any measurable outcome and (S)tudy design – any study design.

## Method

### Search strategy

We performed a systematic review of the literature, developed a priori, to identify case reports, clinical reports or clinical studies involving complement inhibiting therapies in patients with SLE. Keywords were identified and search terms used were: “LUPUS ERYTHEMATOSUS, SYSTEMIC” OR “systemic lupus erythematosus” (title, abstract) OR “lupus” (title, abstract) AND exp. “COMPLEMENT INACTIVATING AGENTS” OR “complement inhibitor” (title) OR “complement inhibition” (title) OR “eculizumab” (title) OR “soliris” (title) OR “avacopan” (title).

This search strategy was applied to the search engines Ovid MEDLINE and EMBASE from 2000 to present, this was intended to capture all patients who were treated with complement inhibition (first complement inhibition therapy, eculizumab, was approved for treatment of PNH in 2007). Results were filtered based on the availability of full text English language and all ages, sexes and ethnicities of patients were included. The search was conducted by FB on 17th May 2019.

### Patient population

Patients were identified using the PICOS process – (P)articipants – all ages, sexes and ethnicities included, (I)ntervention – those who received complement inhibition therapy for their SLE, (C)omparison – before and after complement inhibition therapy, (O)utcome – any measurable outcome and (S)tudy design – any study design. The inclusion criteria for this study was all patients with SLE who had received complement inhibition therapy as treatment for their SLE with any age, sex or ethnicity. Patients were excluded if eculizumab was administered for a condition unrelated to their SLE or if there was no data available on the outcome. Outcome was defined as response to eculizumab therapy - favourable outcome was defined as resolution of the symptoms that led to treatment, discharge from hospital or recovery of renal function. Unfavourable outcome was defined as continuation of symptoms that lead to treatment or death. Adverse effects were defined as any negative effect that occurred during eculizumab therapy unrelated to their primary SLE. Where no data were available for a particular outcome characteristic, this was excluded from the analyses.

### Data collection

Studies were identified through the above criteria (performed by FB, checked by RW) and were analysed independently by two reviewers (RW and LO) by abstract screening. Each manuscript was evaluated using full text to establish the indication for eculizumab treatment, previous medications, demographics, protocol used and outcome. Discrepancies on clinical features were resolved by consensus (RW and LO). Data were summarised qualitatively due to a lack of quantitative data associated with the studies.

### Ethics

In accordance with NHS Health research authority guidance, ethical approval was not required as this systematic review included extracting data from studies that present anonymised individual patient data.

## Results

### Search results

**Fig. 1:**
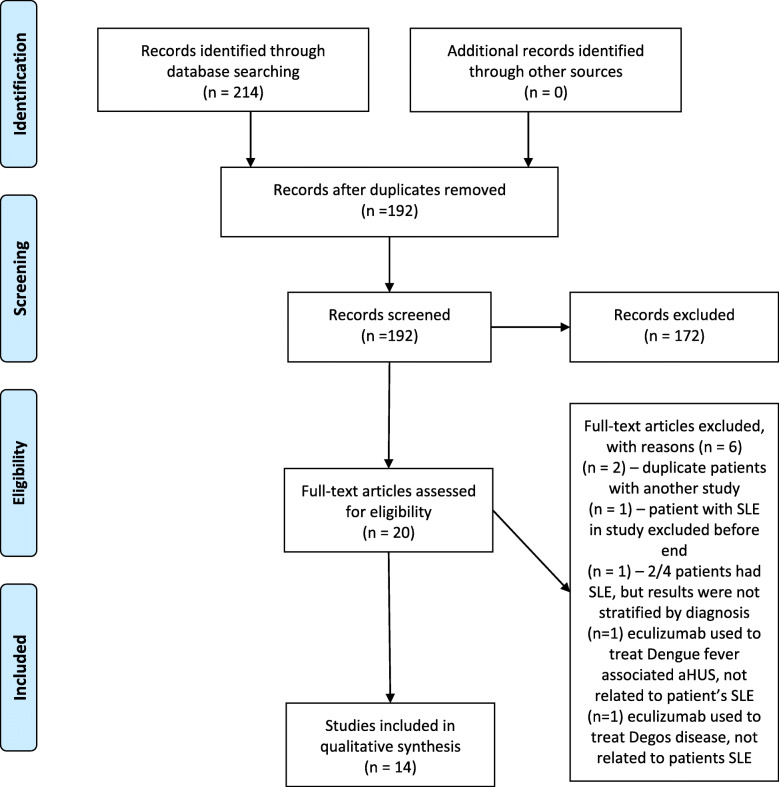
PRISMA 2009 Flow Diagram for search strategy

The search strategy identified 214 records using the Ovid MEDLINE and EMBASE search engines, following removal of duplicates, 192 abstracts were screened. Of these, 172 were declared not suitable (174 by RW, 172 by LO) and 20 full-text articles were accessed for detailed inspection. Six further articles were excluded at this stage (involving 7 patients): two involved the same patient cases duplicated between published journals and published conference abstracts, one study reported a patient with SLE who was excluded before the end of the study, one study looked at four patients, two of whom had lupus but the results were not stratified by diagnosis and so were uninterpretable, one study reported a patient with SLE who was given eculizumab to treat an episode of aHUS caused by Dengue fever [25], and one study reported a patient with inactive SLE who was given eculizumab to treat Degos disease [26]. Therefore, 14 papers were included in the systematic literature review [21, 27–39] (Fig. 1). Inter-rater agreement at the abstract and full article stages were determined by Cohen’s kappa coefficient (0.94 and 1 respectively).

### Patient population

Characteristics of the 14 studies included in the systematic review are summarised in Table 1. Altogether 30 patients were included in the analyses and of these 80% (24/30) were female with a median age of 30 [range 4–59]. Two (7%, 2/28) of the SLE patients included in this study were children (< 18 years) and of those with data available there were six patients that were newly diagnosed (30%, 6/20). Of the patients with a known diagnosis of SLE, three had childhood onset SLE but were adults when eculizumab was administered (21%, 3/14). The studies predominantly came from the United States (70%, 21/30) with the rest from Europe (27%, 8/30) and Brazil (3%, 1/30).

**Table 1:**
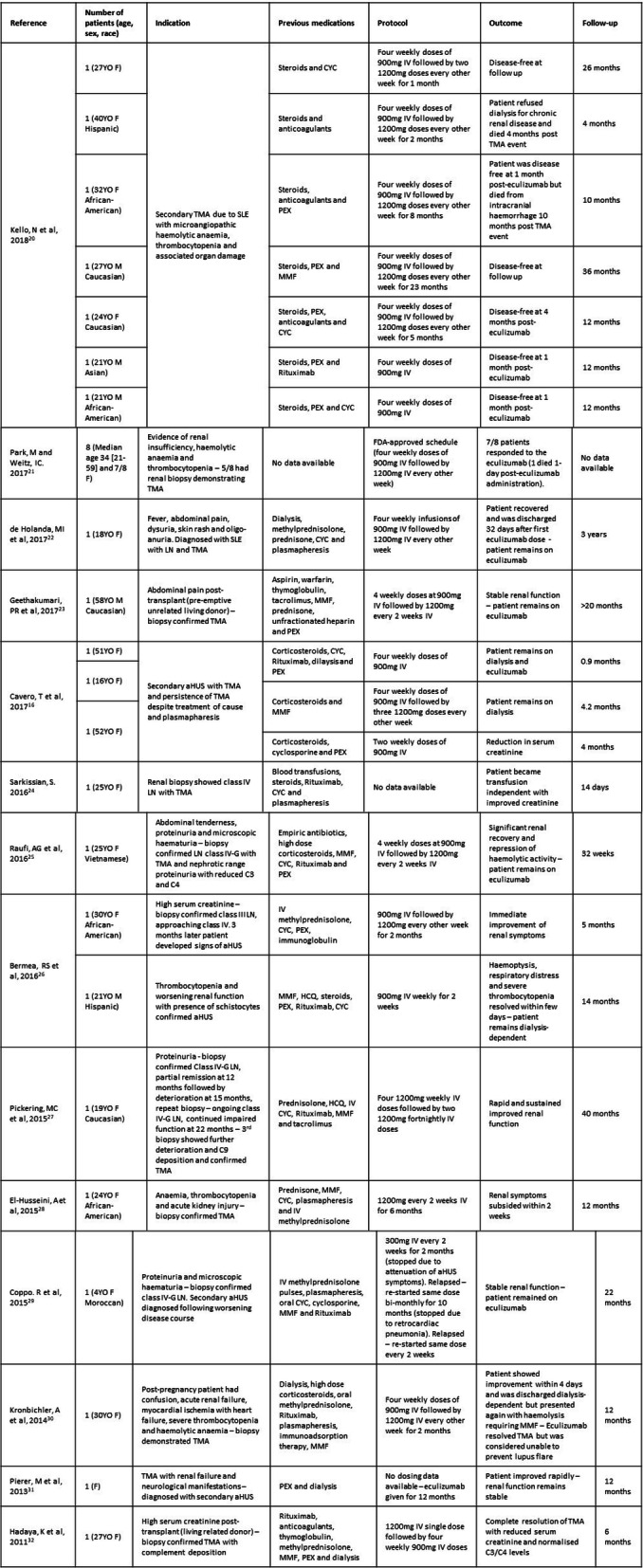
Demographic and clinical data of LN patients treated with eculizumab. aHUS- atypical haemolytic uraemic syndrome, CYC- cyclophosphamide, F- female, GI- gastrointestinal, G- global, HCQ- hydroxychloroquine, IV- intravenous, LN- lupus nephritis, M- male, MMF- mycophenolate mofetil, PEX- plasma exchange, TMA- thrombotic microangiopathy, YO- year old

### Indications

The indication for eculizumab therapy in all cases was a diagnosis of TMA secondary to active LN. The secondary TMA was determined either histologically (66%, 19/30) or by diagnosis of aHUS (a combination of microangiopathic haemolytic anaemia, thrombocytopenia and renal impairment (73%, 22/30)).

Medications attempted prior to eculizumab treatment were varied. However, all patients (where data were available (73%, 22/30)) had previously been treated with corticosteroids and the majority of these had received plasma exchange therapy (77%, 17/22). The most common other treatments used among these patients were: cyclophosphamide (55%, 12/22), mycophenolate mofetil (45%, 10/22) and rituximab (41%, 9/22).

### Eculizumab protocols

The majority of patients (including one paediatric patient aged 16 years old) followed the Food and Drug Administration (FDA)-approved dosing schedule [40], namely four weekly doses of 900 mg IV followed by 1200 mg IV doses every other week (82%, 23/28). However, a proportion of these patients never reached the 1200 mg doses due to rapid remission of disease symptoms (22%, 5/23). The remaining five patients for whom data were available followed slightly different protocols. One patient received a single 900 mg IV dose followed by two weekly 1200 mg doses, one patient received 1200 mg weekly for 4 weeks followed by 1200 mg every 2 weeks. Another patient received 1200 mg every 2 weeks, one patient had a single 1200 mg dose followed by four weekly 1200 mg doses and a final paediatric patient (4 years old) received 300 mg every 2 weeks.

### Outcomes

**Fig. 2:**
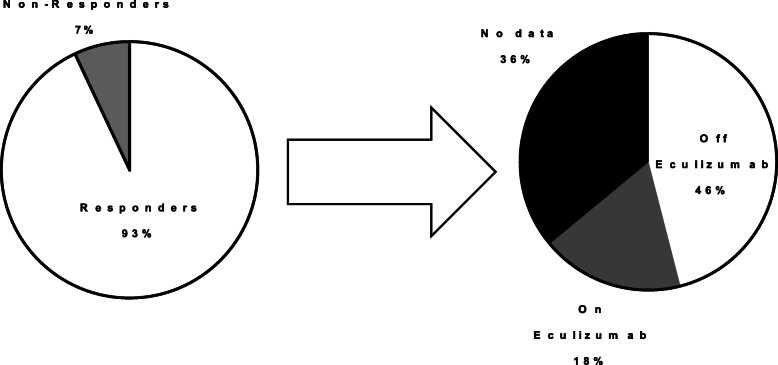
Patient response to Eculizumab therapy. 93% of patients to whom eculizumab was administered responded favourably. Of these 46% were withdrawn from treatment, 18% remained on treatment at follow up and 36% had no data available

The majority of patients had favourable outcomes of eculizumab therapy (93%, 28/30). Favourable outcome was defined as resolution of the symptoms that led to treatment (86%, 24/28), discharge from hospital (82%, 23/28), or recovery of renal function (75%, 21/28) (two of these patients died in a manner reported to be unrelated to eculizumab therapy or TMA, one from an intracranial haemorrhage and one patient declined dialysis). Recovery was rapid in patients who responded where data was available (median 2.5 weeks [range 0.75–16 weeks]). Of the two patients who did not respond, one died within 1 day of eculizumab administration, but the reason was not provided. The other patient was followed up only until his final eculizumab dose (4 weeks) and remained dialysis dependent (Fig. 2).

Of the 28 patients who responded to treatment, 46% (13/28) successfully stopped eculizumab treatment, 36% (10/28) had no data available and 18% (5/28) are still receiving eculizumab treatment (1 patient stopped treatment twice and relapsed requiring repeat doses). There was a short median follow up time of 7 months [range 0.45–40] (Fig. 2).

The majority of patients (90% - 27/30) reported no adverse events in response to eculizumab therapy. One study recorded a patient exhibiting acute pancreatitis, diarrhoea and pneumonia that required 3 stays in hospital over the 6-month course of eculizumab treatment however it was not stated whether this was believed to be directly related to the eculizumab therapy. Another study reported an incidence of nausea without vomiting in a patient on eculizumab and another of nausea with vomiting, however both resolved within 24 h and did not recur during therapy.

## Discussion

The role of the complement cascade in LN is well evidenced; deficiencies in components of the complement cascade can lead to monogenic lupus (C1q, C2 and C4) [14] and low levels of C3 and C4 are assessed in the clinic to monitor disease activity. Further glomerular deposition of complement is almost universally seen in histological analysis. A study of 222 Chinese patients with active LN demonstrated that the alternative complement pathway may be important in the pathogenesis of LN and that factor Bb may be a biomarker for measuring disease activity [18]. Another study of 30 LN patients showed increased deposition of C9 in glomerular biopsy tissue in patients with worse disease compared to those with a more mild phenotype suggesting that the terminal complement complex (C5b-9) may be a suitable biomarker for LN disease activity [41].

Despite the above evidence that complement is important in the pathogenesis of LN, current therapies for LN are broad spectrum immunosuppressants and complement inhibitors are not routinely used. Eculizumab is both effective and safe in the treatment of aHUS [42]. It is well recognised that LN patients can develop secondary aHUS and TMA. However, despite the efficacy of eculizumab in aHUS patients this is not used as first line treatment in these patients. This review summarises case reports and studies that suggest that the use of eculizumab in LN complicated by TMA can be beneficial to patients with excellent response rates described after a short course of treatment. Further only 3 patients reported adverse events during eculizumab therapy suggesting that the safety profile of the drug is good. Eculizumab may have a role in refractory LN but this systemic literature review did not reveal any reported cases and the cost of this drug may be prohibitive [43].

TMA is seen in a subset of LN patients (1–4%) [11] and a small study of 11 patients demonstrated that 60% of these had complement regulatory protein mutations previously associated with aHUS [44]. No other studies have analysed the association between SLE patients with complement mutations and the prevalence of TMA however this is an area that requires more investigation.

Further mechanistic work is required to determine whether it is possible to identify patients with LN who may benefit from these medications as it may be possible to stratify patients. Correlating clinical phenotype with markers of complement activation would help to determine which patients should receive complement inhibition, at what point in disease course, and when is it safe to stop. The use of adjunctive treatment such as Eculizumab to current management protocols may improve the suboptimal renal outcomes in patients with LN.

One limitation of this study is that data related to the serological or non-renal parameters of disease is only available for four of the 30 patients included. This data may provide increased evidence on the risks or benefits of using eculizumab for LN-associated TMA in SLE patients.

## Conclusion

This systematic review has demonstrated that clinically, eculizumab is a treatment option in secondary aHUS or TMA in patients with LN. Patients with refractory LN may benefit from adjunctive treatment with complement inhibition but further studies are required to define its role in this disease.

## Data Availability

Not applicable.

## Abbreviations

aHUS: Atypical haemolytic uraemic syndrome
DMARDs: Disease modifying anti-rheumatic drugs
dsDNA: Double stranded DNA
LN: Lupus nephritis
PNH: Paroxysomal nocturnal haemoglobinuria
SHARE: Single Hub and Access point for paediatric Rheumatology in Europe
SLE: Systemic lupus erythematosus
STEC-HUS: Shiga toxin-producing *Escherichia coli* - haemolytic uraemic syndrome
TMA: Thrombotic microangiopathy
